# Genome Sequence of *Halomonas* sp. Strain MCTG39a, a Hydrocarbon-Degrading and Exopolymeric Substance-Producing Bacterium

**DOI:** 10.1128/genomeA.00793-15

**Published:** 2015-07-16

**Authors:** Tony Gutierrez, William B. Whitman, Marcel Huntemann, Alex Copeland, Amy Chen, Nikos Kyrpides, Victor Markowitz, Manoj Pillay, Natalia Ivanova, Natalia Mikhailova, Galina Ovchinnikova, Evan Andersen, Amrita Pati, Dimitrios Stamatis, T. B. K. Reddy, Chew Yee Ngan, Mansi Chovatia, Chris Daum, Nicole Shapiro, Michael N. Cantor, Tanja Woyke

**Affiliations:** a School of Life Sciences, Heriot-Watt University, Edinburgh, United Kingdom; b Department of Microbiology, University of Georgia, Athens, Georgia, USA; c DOE Joint Genome Institute, Walnut Creek, California, USA

## Abstract

*Halomonas* sp. strain MCTG39a was isolated from coastal sea surface water based on its ability to utilize *n*-hexadecane. During growth in marine medium the strain produces an amphiphilic exopolymeric substance (EPS) amended with glucose, which emulsifies a variety of oil hydrocarbon substrates. Here, we present the genome sequence of this strain, which is 4,979,193 bp with 4,614 genes and an average G+C content of 55.0%.

## GENOME ANNOUNCEMENT

*Halamonas* sp. strain MCTG39a from a sea surface water sample collected off the coast of California was isolated by enrichment with *n*-hexadecane as the sole carbon source (K. Salek, E. Sheehan, and T. Gutierrez, unpublished results). The strain produces a surface-active (amphiphilic) exopolymer, which emulsifies a range of hydrocarbons and increases their bioavailability for biodegradation. Strain MCTG39a is a strictly aerobic and motile, rod-shaped marine bacterium that degrades hydrocarbons. It produces an exopolymeric substance (EPS) with strong emulsifying qualities against a range of hydrocarbons.

Here, we report the genome sequence of *Halomonas* sp. strain MCTG39a. Genomic DNA was isolated, and the sequence was generated at the Department of Energy (DOE) Joint Genome Institute (JGI) using Pacific Biosciences (PacBio) technology. A Pacbio SMRTbellTM library was constructed and sequenced on the PacBio RS platform, which generated 247,476 filtered subreads totaling 781.1 Mbp. All general aspects of library construction and sequencing performed at the JGI can be found at http://www.jgi.doe.gov. The raw reads were assembled using HGAP (version: 2.1.1) (1). The final draft assembly produced 2 scaffolds containing 2 contigs a total of 5.0 Mbp in size with input read coverage of 174.6×.

Project information is available in the Genomes OnLine Database (2). Genes were identified using Prodigal (3), followed by a round of manual curation using GenePRIMP (4) as part of the JGI’s microbial annotation pipeline (5). The predicted coding sequences (CDSs) were translated and used to search the National Center for Biotechnology Information (NCBI) nonredundant database and the UniProt, TIGRFam, Pfam, KEGG, COG, and InterPro databases. The tRNAScanSE tool (6) was used to find tRNA genes, whereas rRNA genes were found by searches against models of the rRNA genes from SILVA (7). Other noncoding RNAs, such as the RNA components of the protein secretion complex and the RNase P, were identified by searching the genome for the corresponding Rfam profiles using Infernal (http://infernal.janelia.org). Additional gene prediction analysis and manual functional annotation was performed within the Integrated Microbial Genomes–Expert Review (IMG ER) platform (http://img.jgi.doe.gov) developed by the Joint Genome Institute, Walnut Creek, CA, USA (8).

The complete genome sequence length is 4,979,193 bp with a G+C content of 55.0%. The genome contains 4,614 genes (4,522 protein-coding genes) with functional predictions for 3,957 of them. A total of 92 RNA genes were detected. Other genes, characteristic for the genus, are given in the IMG database (8). This genome sequence is expected to provide great insights into the unusual life style of this organism.

### Nucleotide sequence accession numbers.

The draft genome sequence of *Halomonas* sp. strain MCTG39a obtained in this study was deposited in GenBank as part of BioProject no. PRJNA224116, with individual genome sequences submitted as whole-genome shotgun projects under the accession no. JQLV00000000.
